# A true trilobed gallbladder, an extremely rare gallbladder malformation

**DOI:** 10.1016/j.radcr.2026.01.012

**Published:** 2026-02-05

**Authors:** Bewuketu Kefyalew, Alamirew Beneberu

**Affiliations:** Department of Radiology, Myung sung Christian Medical College, Jackros - Salite Mehret Road, 17293 Addis Ababa, Ethiopia

**Keywords:** Gallbladder malformation, Trifoliate gallbladder, Trilobed gallbladder, Triple gallbladder

## Abstract

Congenital anomalies of gallbladder are very rare and a true trilobed anomaly of the gallbladder is extremely rare that there is only a single case report in the world literature. This case presents the second case of a true trilobed gallbladder, a different entity from the triple gallbladders, where 3 different lobes of the gallbladder are interconnected to each other by a narrow neck like communication and drains to a single cystic duct through one of the lobes.

## Introduction

True trilobed gallbladder is an extremely rare congenital malformation where the gallbladder has 3 distinct cystic sacs with interconnection via a narrow neck like structure and drain to the cystic duct through one of the lobes or sacs. There is no accessory duct or common communication with the cystic duct.

There is only a single case report of true trilobed gallbladder in the English literature as far as our knowledge [1]. There are few case reports published as a trilobed gallbladder and trifoliate gallbladder as well as triple gallbladder itself but all were similar triple gallbladders [[2], [3], [4], [5], [6]]. A Triple gallbladder is a congenital anomaly where there is persistence or incomplete regression of the rudimentary bile ducts [3]. These different gallbladders are separated by septa, and have different ways of drainage system ranging from 3 different cystic ducts in type I to a common cystic duct as in type III [[2], [3], [4],^6^].

Here we present the second case of a true trilobed gallbladder with associated choledochal cyst.

## Case presentation

A 50 years old male patient came to our emergency department for the complaint of right upper quadrant abdominal discomfort of 2 days. He had associated loss of appetite and few episodes of vomiting. Otherwise, he had no fever, chills or rigors or change in color of eyes, skin or urine. No recorded history of known chronic illness like DM, HTN or malignancy. The patient had other 2 episodes of similar manifestations, RUQ pain and vomiting about a year back which was severe enough and he was admitted in nearby private health care center for the diagnosis of acute cholecystitis.

The patient’s vital signs were within normal range. Physical examination was none revealing except mild right upper quadrant tenderness. Laboratory investigation showed elevated liver enzymes (GOT=249U/L, SGPT=291U/L, GGT=225U/L, total Bilirubin = 2.9mg/dl and direct Bilirubin = 0.7mg/dl). Radiology department was consulted to do ultrasound for acute abdominal pain.

## Imaging findings

**Fig. 1:**
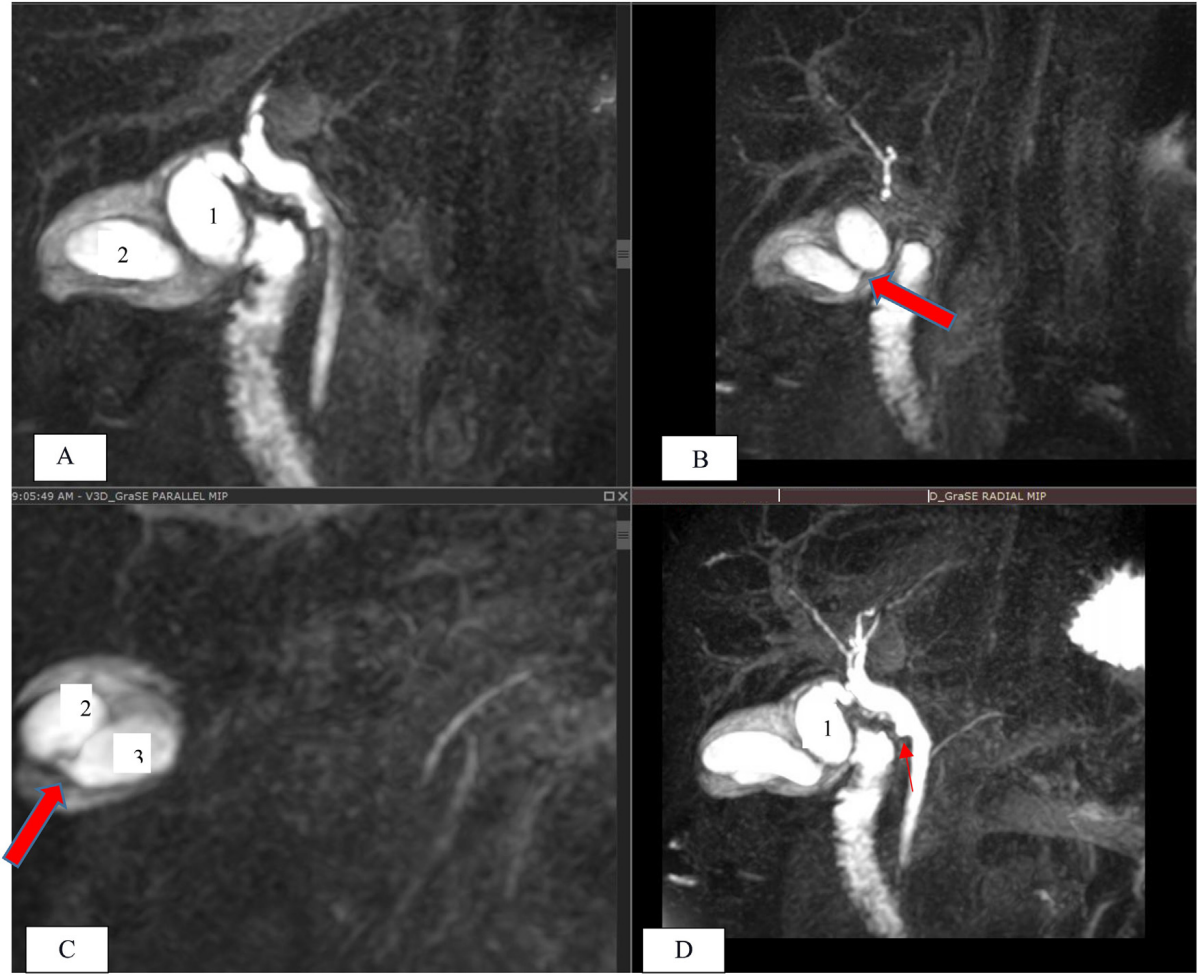
Coronal GraSE images (A–C) and V3D MIP image (D), at different levels: (A) at the level of the fundus of the stomach, shows the primary lobe (*labeled 1*) draining to the cystic duct and (B) at the level of the second part of duodenum showing the communication of the second lobe with the primary sac (*large solid red arrow* in b). (C) Shows the third lobe or sac (*labeled 3*) postero-inferiorly from the second lobe or sac (*labeled 2*) with a narrow inter-communication (*large red arrow in c*) and (D) V3D MIP image showing the dilated CBD with a small outpunching at the mid CBD (*small red arrow in d*), and the 2 lobes interconnected to each other and draining to the cystic duct through the primary (labeled 1) lobe. The third lobe is also visualized inferiorly and partially obscured by the second lobe. Note the peri-cholecystic edema and thickening.

**Fig. 2:**
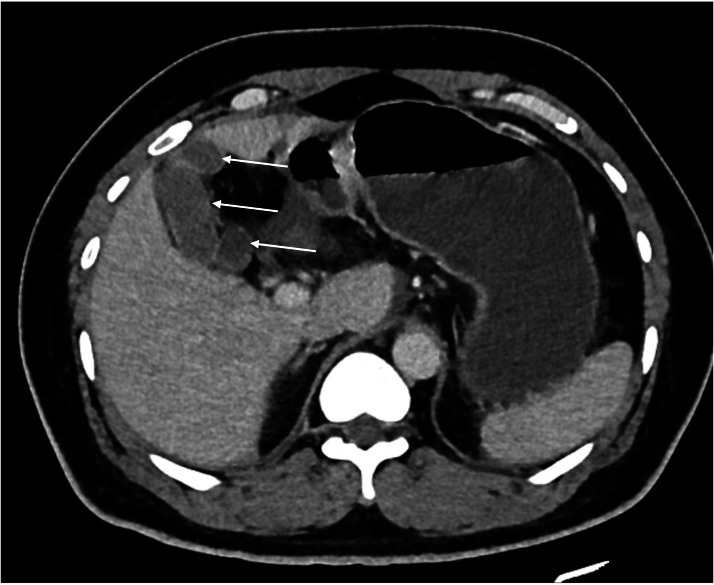
Axial portal venous phase abdomeninal CT of the same patient at the level of T11 taken 10 months prior from the MRCP shows 3 distinct lobes of the gallbladder (arrows) draining to the single cystic duct through the medial lobe.

On sonographic examination: the cystic anomaly was not detected but dilated CBD of up to 9mm was evident with no gallstones in the gallbladder or biliary tree. Consecutively MRCP was done as a further workup for unexplained dilated CBD. MRCP showed dilated CBD with smooth tapering without intrahepatic biliary duct dilatation, focal outpouching of the middle CBD, and 3 distinct gallbladder sacs interconnected to each other by a narrow neck. These gallbladder sacs or lobes finally drain in to a single cystic duct from medial lobe (primary sac) (Fig. 1). Diffuse edematous wall thickening was also noted suggesting acute cholecystitis. Also note the 3 different gallbladder lobes (white arrows in Fig. 2.) interconnected with a narrow neck (not shown) in the CT scan done about 10 months prior from the MRCP (Fig. 2).

Patient started medical treatment for the diagnosis of acute cholecystitis secondary to trilobed GB with mixed type I and type II choledochal cysts and discharged improved.

## Discussion

Gallbladder anomalies are rare congenital anomalies [4]. Multiple gallbladders are rare variants of gallbladder anomalies due to failure in different staged of normal organogenesis of the biliary tree during fetal life [7]. These anomalies can occur due to early divisions, late division, abnormal migration or failure in canalization of the pars cystica (the embryological origin of gallbladder, cystic duct and common bile duct).

These congenital anomalies are asymptomatic in most of the patients unless complicated by acute cholecystitis with or without gallstone formation or found incidentally by imaging for other reasons [2,3]. Our case shows a gallbladder with 3 lobes with a narrow neck like communication to each other and drain to the single cystic duct via the primary lobe. An associated dilated CBD with focal outpouching at the middle segment is also evident suggesting mixed type I and type II choledochal cysts. The circumferential thickening of the gallbladder wall with peri-cholecystic edema are imaging signs of acute cholecystitis as a complication of the trilobed gallbladder probably from biliary stasis.

There is only a single case of true trilobed GB reported previously, although 22 cases of triple gallbladder are reported as trilobed, tifoliate and triple gallbladders [[2], [3], [4], [5],^7^]. This makes our case to be the second case of a true trilobed gallbladder in English literature.

## Conclusion

Our case shows an exceptionally rare variant anatomic malformation of the gallbladder, a true trilobed gallbladder, a distinct anatomic entity from the different subtypes of triple gallbladders. An associated mixed type I and II choledochal cyst makes this case as an exceedingly rare case of such type. Knowledge of such anatomic variant can help for early diagnosis and treatment of acute or chronic complications.

## Limitation

Absence of previous case reports and research on the condition makes difficult to comment on the long term complications and treatment options of the disease.

## Patient consent

Informed and written consent was taken from the patient after thorough discussion about the case and case report.
